# Reframing cancer cachexia: the vagal brain–liver axis as a novel neuro-metabolic target

**DOI:** 10.1038/s41392-025-02483-6

**Published:** 2025-11-11

**Authors:** Daoyan Wei, Xinwen Lin, Robert S. Bresalier

**Affiliations:** 1 Departments of Gastroenterology and Hepatology, The University of Texas MD Anderson Cancer Center, Houston, TX USA; 2 Gastrointestinal Medical Oncology, The University of Texas MD Anderson Cancer Center, Houston, TX USA

**Keywords:** Cancer therapy, Cancer metabolism

In a recent study published in Cell (2025),^1^ Garrett et al. reframed cancer-associated cachexia (CAC) as a neuro-metabolic disorder driven by vagal brain–liver signaling. Non-invasive vagal blockade restored metabolism, preserved muscle, and prolonged survival, positioning neuromodulation as a transformative strategy in supportive oncology.

**Fig. 1:**
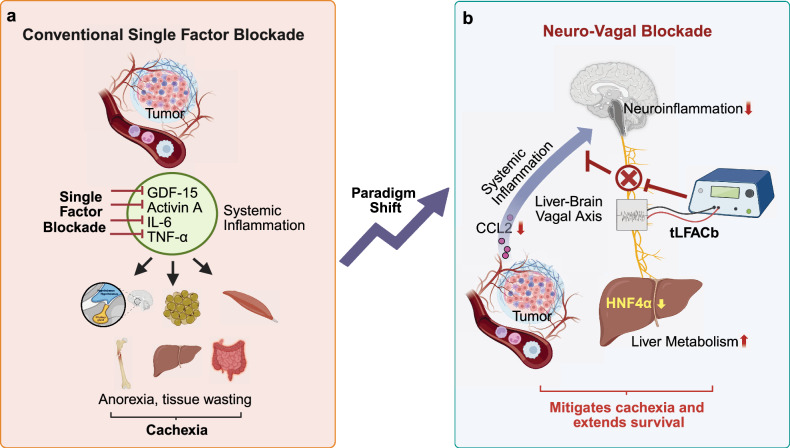
Conceptual shift in cancer cachexia therapy. **a** Cachexia has traditionally been viewed as a cytokine-driven inflammatory syndrome, yet single-cytokine blockade yields minimal benefit. **b** Garrett et al. reframe it as a neuro-metabolic disorder, wherein tumor-induced inflammation (notably CCL2) triggers vagal neuroinflammation, disrupting brain–liver signaling and metabolism. Therapeutic vagal blockade (tLFACb) mitigates cachexia and prolongs survival, highlighting neuromodulation as a novel management strategy. Figure was created in BioRender. XL (2025)

CAC is a multifactorial, systemic inflammatory, and lethal syndrome characterized by involuntary weight loss, skeletal muscle wasting, adipose tissue depletion, anorexia, fatigue, and profound metabolic alterations. It severely undermines tolerance to therapies, erodes quality of life, and is a major driver of morbidity and mortality. CAC affects up to 85% of patients with advanced cancers, particularly pancreatic and lung cancer, and accounts for nearly one-third of cancer-related deaths.^2^ Despite its prevalence and devastating impact, CAC remains largely untreatable; no FDA-approved therapies effectively prevent or reverse the syndrome. Attempts to intervene “one node at a time”—for example, through blockade of individual cytokines or stimulating appetite—have provided only transient or partial relief. GDF15- or IL-6–targeted strategies exemplify this limitation: modest improvements in appetite or weight, have not consistently translated into survival benefit^3^ (Fig. 1a). These shortcomings highlight the need for circuit-level interventions that address how tumors reprogram whole-body physiology.

A landmark study by Garrett et al.^1^ provides compelling evidence that tumor-driven systemic inflammation distorts vagal signaling, disrupting brain–liver communication and depleting hepatic HNF4α, a master transcriptional regulator of metabolism. This cascade drives anorexia, muscle wasting, and other cachexia hallmarks. Crucially, blockade of the right cervical vagus nerve—via surgical, chemical, or non-invasive neuromodulatory approaches—attenuated cachexia symptoms, improved chemotherapy tolerance, and, most importantly, extended survival in murine pancreatic and lung cancer models (Fig. 1b). By reframing cachexia as a neuro-metabolic disorder, this work redefines the vagus nerve from a passive bystander of sickness to an active driver and therapeutic target, opening new avenues for neuromodulatory interventions and marking a paradigm shift in supportive oncology.

The greatest strength of this study lies in its mechanistic dissection of vagal dysfunction. Electrical recordings from the hepatic branch of the subdiaphragmatic vagus nerve in murine CAC models (orthotopic KPC and LLC) revealed increased signal ‘noisiness’ (elevated RMS values) coupled with reduced amplitude. These impairments were linked to neuroinflammation in the vagal dorsal motor nucleus (VDMN), marked by increased macrophage infiltration driven by the chemokine CCL2.

Genetic or pharmacologic inhibition of CCL2 (via Bindarit) significantly reduced neuroinflammation, normalized vagal tone, lowered systemic acetylcholine (ACh), and ameliorated cachexia parameters, highlighting CCL2 as a driver of vagal dysregulation. Notably, these changes preceded significant weight loss, positioning vagal dysfunction as an initiating rather than secondary event in CAC pathogenesis.

Downstream, aberrant ACh signaling suppressed hepatic hepatocyte nuclear factor 4α (HNF4α) expression, disrupting key metabolic programs essential for systemic nutrient balance. Hepatic HNF4α loss induced upregulation of LCN2, a pro-cachectic, pro-inflammation, appetite-suppressing factor.^4^ Hepatocyte-specific HNF4α knockdown recapitulated cachexia-like wasting in healthy mice, whereas vagotomy restored HNF4α, normalized amino acid metabolism, and lowered circulating LCN2. These findings identify liver as a key downstream effector of vagal dysfunction, reinforcing a brain–vagus–liver–metabolism axis as a unifying framework for CAC pathogenesis.

The most striking results emerged from interventional studies. Right cervical vagotomy in cachectic mice preserved body weight, lean muscle and fat mass, improved grip strength, reduced anorexia- and depression-like behaviors, and prolonged survival despite unchanged tumor burdens. Vagotomy also synergized with cisplatin, further extending survival. Serum cytokine profiling confirmed reduced CCL2 and other pro-inflammatory mediators, supporting the role of vagal signaling in CAC. These results challenge the entrenched assumption that tumor control is the only path to mitigating cachexia.

Equally exciting was the demonstration that low-frequency alternating current block (LFACb), delivered via implanted electrodes or a non-invasive adhesive transcutaneous patch (tLFACb), recapitulated the benefits of vagotomy. Daily 30-minute sessions preserved weight and muscle, improved behavior, and significantly extended survival in cachectic mice, with further gains when combined with chemotherapy. Proof-of-concept swine studies confirmed feasibility and safety, showing robust efficacy without any adverse cardiovascular or tissue effects. This non-invasive approach offers a clinically viable neuromodulatory strategy.

While groundbreaking, this study leaves several intriguing questions for future investigation. *First*, the precise pathway linking vagal ACh signaling to suppression of hepatic HNF4α remains unresolved. It is unclear which hepatocyte ACh receptor subtypes (muscarinic vs. nicotinic) and downstream pathways (e.g., Ca²⁺-dependent, PKC, MAPK, PI3K/AKT) mediate this effect. In addition, how CCL2-driven VDMN neuroinflammation alters vagal tone and hypothalamic feeding circuits, how hepatic HNF4α regulates LCN2 expression, and whether HNF4α also controls other hepatokines that promote wasting remain open questions.^5^ Additionally, many immune cells also express ACh receptors, raising the possibility that tumor immunity may likewise be modulated by vagal signaling in this process, which warrants further investigation (Fig. 1b).

*Second*, CAC is driven by multiple mediators (GDF15, IL-6, TNF-α, myostatin), and this study also observed elevations in IL-1β, MIP2, IL-2, and CXCL10. Such redundancy may explain why Bindarit or vagotomy alone did not fully reverse the syndrome. Combining tLFACb with anti-GDF-15 therapy (e.g., ponsegromab) or anti-inflammatory agents may yield additive benefit. Notably, synergy with immunotherapies is especially intriguing, given that cachexia-induced metabolic dysfunction represents a major barrier to antitumor immunity.

*Third*, vagotomy reduced systemic inflammation without affecting tumor growth or innervation. How this anti-inflammatory effect arises, particularly in models lacking direct vagal innervation (e.g., the subcutaneous LLC model), remains unclear. More representative models (orthotopic, spontaneous, PDX) will be needed to probe tumor–nerve interactions and ACh signaling within the tumor microenvironment.

*Fourth*, it remains unresolved whether vagotomy influences skeletal muscle and adipose wasting directly through ACh signaling or indirectly via reduced tissue inflammation.^4^

*Fifth*, the optimal timing for vagal blockade—preventive vs. therapeutic—has yet to be defined. Biomarkers such as serum CCL2, ACh, LCN2, and hepatic metabolomic signatures may aid patient selection and response monitoring.

*Finally*, although murine and swine studies suggest safety, translating vagal blockade into patients raises concerns given the critical roles of vagus nerve in cardiovascular, gastrointestinal, and immune regulation. Early-phase trials of tLFACb in PDAC and NSCLC should therefore prioritize feasibility, safety, and tolerability, while assessing endpoints such as muscle preservation, chemotherapy dose intensity, patient-reported outcomes, and survival.

Together, Garrett et al. deliver a landmark study reframing CAC as a disorder sustained by vagal brain–liver circuitry rather than a passive consequence of tumor burden. By showing that targeted right vagal blockade, especially via a feasible, non-invasive transcutaneous approach, preserves muscle mass, enhances chemotherapy response, and most importantly, extends survival, this work challenges long-held paradigms and opens a bold new direction in supportive oncology. Additionally, vagal neuromodulation may extend to other wasting syndromes, including heart failure, chronic obstructive pulmonary disease, and sepsis, where cachexia-like mechanisms overlap.

The road ahead requires mechanistic refinement, biomarker validation, and rigorous translational testing. Yet, the conceptual leap—rewiring neural circuits to combat cachexia rather than simply feeding the patient or targeting single cytokines—offers a transformative shift in CAC management.
